# Modulation of Gut Microbiota by Whole Encapsulated Brown Seaweed (*Himanthalia elongata*) in Overweight Subjects: A Randomized Double-Blind Placebo-Controlled Trial

**DOI:** 10.3390/nu17122047

**Published:** 2025-06-19

**Authors:** Aroa Lopez-Santamarina, Alejandra Cardelle-Cobas, Alicia del Carmen Mondragon, Alberto Cepeda, Jose A. Rodriguez, Jose Manuel Miranda

**Affiliations:** 1 Department of Analytical Chemistry, Nutrition and Food Science, School of Veterinary Sciences, University of Santiago de Compostela, 27002 Lugo, Spain; aroa.lopez.santamarina@usc.es (A.L.-S.); alejandra.cardelle@usc.es (A.C.-C.); alicia.mondragon@usc.es (A.d.C.M.); alberto.cepeda@usc.es (A.C.); 2 Área Académica de Química, Universidad Autónoma del Estado de Hidalgo, Carr. Pachuca-Tulancingo Km. 4.5, Mineral de la Reforma 42184, Mexico; josear@uaeh.edu.mx

**Keywords:** prebiotic, gut microbiota, *Himanthalia elongata*, short-chain fatty acids, randomized controlled trial

## Abstract

**Background/Objectives**: Brown seaweeds, such as *Himanthalia elongata*, are a promising source of dietary fiber. However, in vivo evidence regarding the effects of *H. elongata* intake on the human gut microbiota remains limited. This study aimed to evaluate the impact of daily *H. elongata* consumption on the gut microbiota composition and short-chain fatty acid production in overweight adults. **Methods**: A randomized, double-blind, placebo-controlled trial was conducted in which 10 overweight adult participants consumed 2 g/day of whole *H. elongata* for 30 days. Fecal samples were collected before and after the intervention for 16S rRNA sequencing and short-chain fatty acid analysis. Dietary intake was evaluated using a 24 h recall and a 3-day dietary record. Nutritional assessment was performed to determine habitual macronutrient consumption. **Results**: Baseline dietary analysis revealed an imbalanced macronutrient profile characterized by high intakes of total and saturated fats and protein, along with low carbohydrate and fiber consumption. In addition, 50% of the participants were obese, and 50% were overweight based on the BMI. Notable changes in the gut microbiota composition were observed after the intervention, including increases in short-chain fatty acid-producing species, such as *Parabacteroides distasonis*, *Bacteroides eggerthii*, *Bacteroides uniformis*, and *Bacteroides obeum*. **Conclusions**: This study provides the first clinical evidence in humans that whole *H. elongata* can beneficially modulate the gut microbiota composition. These results support the potential use of this seaweed as a functional prebiotic ingredient in dietary strategies aimed at enhancing gut health.

## 1. Introduction

Dietary fiber plays a fundamental role in intestinal transit, satiety regulation, blood glucose control, and lipid metabolism, and its fermentation in the colon contributes to the mucosal health, modulation of inflammation, and maintenance of gut barrier integrity [1,2]. A fiber-rich diet is associated with a lower risk of obesity, type 2 diabetes, cardiovascular disease, and certain cancers [3,4,5]. Moreover, dietary fiber influences the composition and activity of the gut microbiota, favoring the growth of beneficial bacteria such as *Bifidobacterium* and *Lactobacillus* species [6].

Maintaining a balanced, diverse, and resilient gut microbiota (GM) is crucial for supporting overall health and well-being [7]. When this bacterial homeostasis is disturbed, the host health might be affected in several ways [8]. Among other contributors to GM stability, diet, especially the consumption of nondigestible carbohydrates, plays a prominent role. These chemicals contribute to the microbial milieu by stimulating the production of beneficial compounds such as short-chain fatty acids (SCFAs) [9]. SCFAs contribute to several important processes, including energy regulation, hormone secretion in the gut, cholesterol metabolism, and the reduction in inflammation, all of which are vital for maintaining human health [10]. However, the impact of nondigestible carbohydrates on the GM varies depending on their specific characteristics, such as molecular weight, monosaccharide composition, and glycosidic linkages [11].

Despite these benefits, the fiber intake in Western countries remains consistently below the recommended levels. For instance, in Europe and North America, the average daily intake ranges between 15 and 20 g, far from the 25–38 g/day recommended by most dietary guidelines [12]. This deficiency is partly attributed to the increased consumption of ultra-processed foods and the reduced intake of whole grains, fruits, and vegetables, contributing to adverse metabolic outcomes and impaired gut microbiota profiles [13].

In the context of obesity, low fiber intake exacerbates the proinflammatory and dysbiotic state associated with excess adiposity. Increasing the dietary fiber intake in overweight and obese individuals has been shown to improve the microbial diversity and metabolic markers [14].

In Western diets, most nondigestible polysaccharides are derived from the cell walls of terrestrial plants [15]. However, with the rise in global population growth and increasing agricultural yields, there is a growing need to identify new, sustainable sources of dietary fiber [16,17]. Seaweeds have gained attention as a promising alternative. They grow quickly and do not require farmland, freshwater, or fertilizers [18] while also offering environmental benefits such as carbon dioxide absorption [19].

Although green seaweeds are rich in beneficial compounds, they are not widely used in the nutraceutical or pharmaceutical industries [20]. Nutritionally, they are high in protein, low in fat, and rich in essential vitamins, minerals, and phytochemicals [20,21]. One group of interest is polyphenols, which constitute 1–5% of the dry weight of green seaweed [22,23] and are known for their antioxidant and antimicrobial properties [24,25]. Interestingly, polyphenols are not absorbed in the small intestine and reach the colon, where they may positively affect the GM composition [26].

To date, most studies examining the effects of brown seaweed on the GM have been conducted in vitro [27,28,29,30,31,32,33]. Notably, most of this research has focused on isolated polysaccharides, with only one study using whole brown seaweed [30].

As whole seaweed contains more bioactive compounds than carbohydrates that can influence the GM, assessing their broader impact is important. The current study aimed to address this gap by evaluating the effects of whole and encapsulated *H. elongata* on the GM of overweight individuals, offering a novel approach compared with previous studies that relied solely on purified extracts. This study is the first in vivo investigation of the effects of whole *H. elongata* on the GM.

## 2. Materials and Methods

### 2.1. Ethics

This study was approved by the Ethics Committee of the University of Santiago de Compostela (approval no. 39/2024) and was conducted in accordance with the principles outlined in the Declaration of Helsinki and other international guidelines for research involving human participants [34]. Before joining the study, all the participants were fully informed of the goals, procedures, possible risks, and expected benefits. Each participant provided written informed consent, confirming their voluntary decision to participate.

To ensure the participants’ privacy, all personal data were handled according to the General Data Protection Regulation and Spanish Organic Law 3/2018 on the Protection of Personal Data and Guarantee of Digital Rights [35]. The collected data were anonymized and securely stored, with access strictly limited to the research purposes. The trial was conducted in June 2024 across two regions in northwestern Spain (Galicia and Asturias).

### 2.2. Participants, Study Design, and Intervention

**Figure 1:**
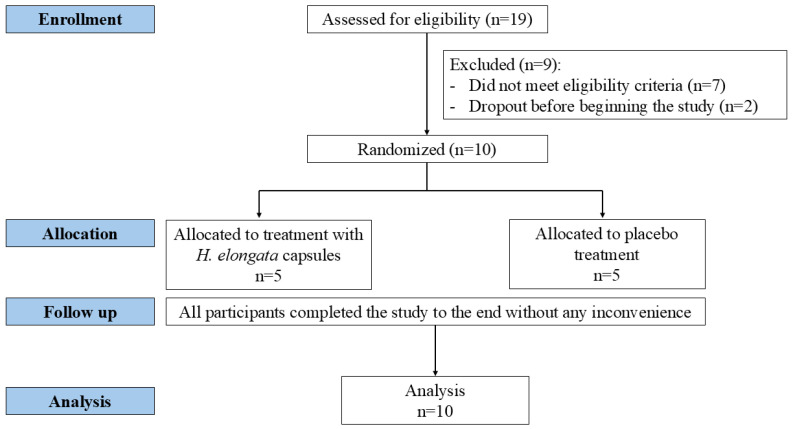
CONSORT flow diagram of participants.

The participants were enrolled in a randomized, double-blind, placebo-controlled trial to evaluate the effects of *H. elongata*, a brown seaweed, on the GM of overweight individuals (Figure 1). Eligible participants were aged between 20 and 65 years; had not taken antibiotics or pharmacological preparations of prebiotics, probiotics, or postbiotics in the previous six months; and had no history of gastrointestinal diseases or disorders. The participants were randomly assigned to one of two groups of 5 subjects (placebo or treatment) using a computer-generated sequence. An independent researcher assigned the subjects to groups to ensure blinding. This study was conducted as an exploratory pilot randomized trial. A formal power calculation was not performed due to its feasibility nature and the limited availability of volunteers. Instead, a pragmatic sample size of 10 participants (5 per group: placebo and treatment) was selected to assess the feasibility, group dynamics, and gather preliminary data.

All capsules (Guinama, Valencia, Spain) were identical in appearance, red, opaque, and made of gelatin and water to prevent the participants from distinguishing between the treatments. Rice starch (Guinama, Valencia, Spain) was chosen as the placebo owing to its neutral taste and texture, as well as its minimal impact on the GM, as it is almost completely absorbed in the small intestine.

Each capsule contained 1 g of either seaweed (treatment) or rice starch (placebo), and the participants were instructed to take two capsules per day for 30 days.

### 2.3. Fecal Sample Collection and Dietary Intake Data

The participants attended the Laboratory of Hygiene, Inspection, and Food Control at the University of Santiago de Compostela (Lugo, Spain), where they received detailed instructions regarding the study’s procedures and duration. During this visit, they provided fresh fecal samples to assess their baseline GM before the intervention (initial time point). After being informed about the study, the participants who chose to participate signed both an informed consent form and a data protection agreement. To assess their dietary intake, two complementary tools were used consistently across all the participants. First, a 24 h dietary recall was conducted during the initial study visit through a personal interview with a trained researcher. This recall captured all the food and beverage intake from the day prior to the baseline visit, providing a standardized snapshot of the participants’ usual diet at the start of the study.

Second, each participant was instructed to complete a 3-day dietary record during the intervention period, covering two weekdays and one weekend day. They were provided with written instructions and guidance to record all foods, beverages, and condiments consumed, including quantities and the preparation methods. This record was used to monitor the habitual intake throughout the intervention under real-life conditions. At the same time, they received a sterile container for collecting a stool sample at the end of the 30-day study period, which they were asked to return to the laboratory for analysis. Specifically, they were asked to note the consumption of water, salt, sugar, and oil, which are not typically reported in regular food records.

In the laboratory, the stool samples were diluted 1:10 in phosphate-buffered saline with 33% glycerol to preserve DNA during freezing and the subsequent analysis.

For nutrient analysis, the dietary data were processed via the “Diet Calculator” free software developed by the Research Center for Endocrinology and Clinical Nutrition (IENVA) [36], which is based on Spanish food composition tables. The results were then compared with the dietary recommendations for adults set by the Spanish Society of Community Nutrition (SENC) [37], the Dietary Guidelines for Americans (DGA) [38], the Food and Nutrition Board (FNB) [39], and the World Organization Health (WHO) [17], allowing for an assessment of how well the participants’ diets aligned with the national nutritional goals.

By combining multiple methods, the study was able to collect comprehensive and reliable dietary data, which was essential for evaluating how the intervention influenced eating habits over time.

### 2.4. DNA Extraction and 16S Ribosomal RNA Amplicon Sequencing

Bacterial DNA was extracted from the fermented samples with the DNA RealPure Spin Food-Stool Kit^®^ (Real, Durviz S.L., Valencia, Spain) according to the protocol established by the manufacturer for fecal samples. A total of 1.2 mL of diluted stool was centrifuged for 7 min at 6100× *g* to obtain a pellet, which was then recovered and used for DNA extraction. The extracted DNA was then quantified via a Qubit™ 4 fluorometer (Invitrogen, Thermo Fisher Scientific, Carlsbad, CA, USA) and a DNA HS Assay Kit (Invitrogen, Thermo Fisher Scientific, Eugene, OR, USA). After quantification, the DNA samples were stored at −20 °C until further analysis. A portion of the samples taken from the in vitro colonic assays was subjected to bacterial DNA extraction and subsequent 16S ribosomal RNA (rRNA) amplicon sequencing, as described previously [40].

### 2.5. Short-Chain Fatty Acid Analysis

The diluted stool was centrifuged for 7 min at 6100× *g*. The supernatants were removed and filtered through 0.2 μm cellulose acetate membranes (Phenomenex, Torrance, CA, USA). Then, 20 μL of each sample was injected into an Aminex HPX-87H column (LC column 300 × 7.8 mm; Bio-Rad, Hercules, CA, USA) operating at 50 °C. The mobile phase, 3 mM sulfuric acid, was flushed through the column at a flow rate of 0.6 mL/min in isocratic mode, and the temperature of the column was maintained at 50 °C throughout the entire run. High-performance liquid chromatography was performed with an Agilent HPLC-PDA system (Waldbronn, Germany) consisting of a binary pump, a degasser, an autosampler, and a column heater coupled to a detector (Infinity 1260 II Diode Array Detector HS; Agilent, Waldbronn, Germany), as previously described by Lopez-Santamarina et al. [40]. SCFAs were quantified by comparing the peak areas with those obtained from external standards.

### 2.6. Statistical and Bioinformatics Analysis

For 16S rRNA amplicon sequencing analysis, raw sequencing reads were downloaded using Torrent Suite software (v.5.20). These files were processed with QIIME 2 software v. 2024.10 and MicrobiomeAnalyst (https://www.microbiomeanalyst.ca/). Amplicon sequence variants (ASVs) were produced using the DADA2 method for quality filtration (Q score > 20), trimming, denoising, and dereplication. Samples with features (taxa) with a total abundance of <10 were normalized via rarefaction. Taxonomy was assigned to ASVs against the Greengenes 13_8 99% operational taxonomic unit (OTU) reference sequences. The samples were rarefied to a sequencing depth of 39,000 reads, and alpha (α) and beta (β) diversities were determined. The Phylogenetic Investigation of Communities by Reconstruction of Unobserved States (PICRUSt2) online Huttenhower Lab Galaxy Server 2.0 was used to predict the functional content of the metagenome. PICRUSt uses an OTU table constructed using the closed benchmark method via QIIME2. The closed benchmark method, the Kyoto Encyclopedia of Genes and Genomes (KEGG) [41], was use to compare each representative OTU sequence with the reference sequences available in each database.

STAMP software (v 2.1.3; “Statistical analysis of taxonomic and functional profiles”) was used to perform ANOVA with the Tukey–Kramer post hoc test to determine significant differences between ASVs and metabolic pathways obtained using QIIME2 and PICRUSt, respectively [42].

## 3. Results and Discussion

### 3.1. Anthropometric Characteristics of the Participants

The anthropometric characteristics of the participants at the baseline are shown in Table 1. The BMI values ranged from 21.3 to 33.9 kg/m^2^, encompassing individuals classified as normal weight, overweight, and obese according to the WHO criteria. The corresponding waist circumference values were consistent with each BMI category, with several participants exhibiting measurements indicative of abdominal obesity. No significant differences in the BMI or waist circumference were observed between the placebo and treatment groups at the baseline.

### 3.2. Assessment of the Dietary Intake

Baseline dietary analysis revealed an imbalanced macronutrient profile characterized by high intakes of total and saturated fats and proteins, along with low carbohydrate and fiber consumption. In terms of nutritional status, 50% of the participants were classified as obese and 50% as overweight according to the BMI.

Table 2 and Table 3 summarize the mean daily intake of energy and macronutrients of the participants, as estimated from a single 24 h dietary recall and a 3-day dietary record. The analysis revealed notable differences between the two dietary assessment methods and important deviations from international dietary recommendations. Although the 3-day dietary record generally yielded higher intake estimates, both methods indicated that the mean daily energy intake was below the estimated energy requirements for adults. This likely reflects underreporting rather than an actual energy deficiency. Single-day recalls are particularly prone to recall bias and international underreporting [43].

The macronutrient distribution was imbalanced across both methods. The protein intake exceeded the upper threshold of national recommendations and slightly surpassed the upper limit set by the European Food Safety Authority (EFSA) [12], although it remained within the safe limits for healthy adults. This suggests a dietary pattern with a relatively high protein intake, which may have implications for long-term metabolic health depending on the protein sources and overall nutrient balance.

The fat intake, particularly saturated fat intake, was consistently high. Both total fat and saturated fat consumption surpassed the recommended upper limits, with the saturated fat intake reaching levels more than double the WHO’s suggested threshold. This pattern is concerning, given the established role of excessive saturated fat in increasing the risk of cardiovascular disease. Conversely, the intakes of monounsaturated and polyunsaturated fats were within acceptable or encouraged ranges, with polyunsaturated fats falling comfortably within the EFSA’s recommended range. Although monounsaturated fats lack specific intake thresholds, they are generally considered beneficial, particularly when saturated fats are replaced [12].

The carbohydrate intake was below the recommended proportion of total energy intake in both the dietary assessments. This, combined with a high-fat intake, indicates a macronutrient distribution skewed toward lipid consumption. Notably, the free sugar intake substantially exceeded the WHO guidelines [17], highlighting the excessive intake of rapidly digestible carbohydrates with low nutritional value.

Furthermore, the fiber intake was found to be insufficient across both assessment methods. These values fell short of the minimum recommended intake, which is particularly relevant in the context of this study, given the important role of dietary fiber in maintaining GM diversity and metabolism. A low fiber intake, in conjunction with high saturated fat and free sugar consumption, may negatively impact the GM composition, potentially reducing the microbial diversity and promoting a proinflammatory profile, as supported by the literature [44].

These dietary profiles are not only misaligned with the current nutritional guidelines but may also contribute to adverse health outcomes, particularly those related to metabolism and health, such as obesity [45].

The cholesterol intake was greater in the 3-day dietary record than in the 24 h recall. Although the current guidelines do not establish a specific upper limit for cholesterol consumption, intakes above the traditionally recommended thresholds remain a concern owing to their association with cardiovascular risk. In line with international recommendations, the SENC [37] advises minimizing the cholesterol intake within a healthy dietary pattern, highlighting the relevance of these values.

With respect to alcohol consumption, no intake was reported in the 24 h recall, whereas the 3-day record indicated limited and infrequent use among the participants.

Table 4 and Table 5 present the micronutrient, mineral, and vitamin intake data. The assessment of the micronutrient intake revealed notable differences between the 24 h dietary recall and the 3-day dietary record, with several nutrients showing either under- or over-estimation, depending on the method. These differences are crucial when evaluating the dietary quality and its potential effects on the GM.

The mineral intake showed variability between the two dietary assessment methods. The calcium intake was higher in the 24 h recall and met the recommended dietary allowance (RDA) for adults, whereas the 3-day record fell slightly below the RDA, potentially owing to daily intake variability or recall bias [39].

The iron intake was below the RDA for premenopausal women in both assessments, although it was adequate for adult men, indicating a possible deficiency risk in certain subgroups.

The magnesium intake was slightly lower than the recommended level for both methods, reflecting a common insufficiency in Western diets [46].

The sodium intake exceeded the recommended upper limit in both dietary assessments, which may contribute to an increased risk of hypertension and related health issues [39].

The potassium intake was higher in the 3-day record, yet both methods indicated intakes below the adequate intake (AI) levels for men and women, suggesting the suboptimal consumption of potassium-rich foods, such as fruits and vegetables.

The iodine intake was higher in the 24 h recall than in the 3-day record, with both methods reporting values above the RDA (150 µg/day), likely reflecting the consumption of iodized salt and seafood. The zinc, selenium, and phosphorus intakes were within or above the recommended levels, with the selenium intake notably elevated in the 3-day record [39].

With respect to vitamins, the vitamin A intake met the RDA in both assessments, although considerable variability was observed between the methods. The carotenoid intake was greater in the 3-day record, suggesting the improved capture of plant-based sources over multiple days. In both methods, the vitamin D intake was insufficient, falling below the RDA (15 µg/day), which underscores the need for fortified foods and supplementation.

Overall, the vitamin C intake was adequate, although it fell below the RDA for some individuals. B-complex vitamins, including thiamin, riboflavin, niacin, and vitamin B6, were generally sufficient, with slightly higher values recorded in the 3-day records.

The folate intake was below the RDA (400 µg/day) in both assessments, which may have implications for gut health and methylation. In contrast, the vitamin B12 intake exceeded the RDA (2.4 µg/day) in both methods, with slightly higher values observed in the 3-day record, supporting adequate intake for optimal neurological function.

### 3.3. Effects of Himanthalia elongata Intake on the Gut Microbiota

#### 3.3.1. Alpha and Beta Diversity

**Figure 2:**
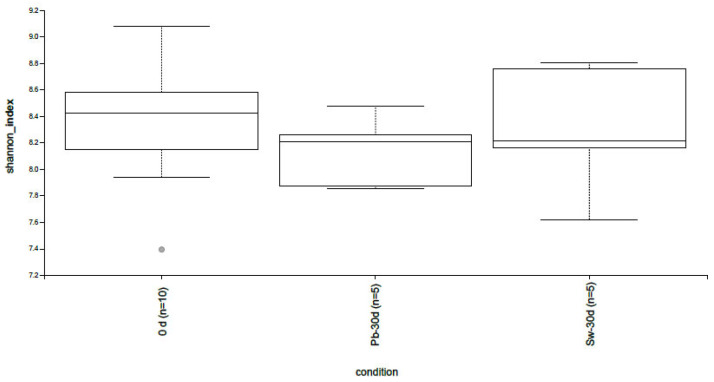
Changes in alpha diversity from baseline (0 d) to the end of the intervention with seaweed (Sw-30 d) and placebo (Pb-30 d).

Alpha diversity analysis using the Shannon index revealed dynamic changes across the three study groups: baseline (0 d), placebo after 30 days (Pb-30 d), and seaweed supplementation after 30 days (Sw-30 d) (Figure 2). At the baseline, the median Shannon index was approximately 8.4, reflecting a relatively high level of microbial diversity, which is consistent with that typically observed in adult populations with no recent antibiotic exposure or severe dietary restrictions [47].

After 30 days, the placebo group (Pb-30d) showed a slight decrease in Shannon diversity (median, 8.1), although this change was not statistically significant. This minor decrease could reflect normal fluctuations in the GM, which are influenced by individual dietary habits or natural variability over time [48]. On the other hand, the group that received *H. elongata* (Sw-30d) presented a modest increase in alpha diversity (median 8.6), but similar to the placebo group, the change was not significant.

These findings suggest that *H. elongata* supplementation may help preserve or modestly increase gut microbial diversity over short periods. Higher microbial diversity is generally associated with greater ecosystem resilience and metabolic versatility, which are linked to improved health outcomes [49]. Specifically, reduced microbial diversity has been correlated with obesity, type 2 diabetes, and inflammatory bowel disease [50].

The trend toward increased alpha diversity following *H. elongata* intake aligns with previous in vitro and in vivo studies that have shown the prebiotic effects of brown seaweed. These effects are largely attributed to the high content of nondigestible polysaccharides, such as alginates, fucoidans, and laminarins, which selectively promote the growth of beneficial gut bacteria [51,52].

This broader distribution indicates that while the seaweed intervention did not lead to a uniform increase in microbial diversity across all the participants, some individuals experienced increases in alpha diversity that exceeded the baseline levels. These individual responses suggest a heterogeneous effect of algae, potentially influenced by host-specific factors such as the baseline GM composition, diet, genetics, or metabolic status. This observation aligns with the findings of Zhang et al. [53], who reported that supplementation with Laminaria japonica led to variable changes in the GM composition and polysaccharide utilization among individuals, highlighting the role of interindividual differences in dietary fiber metabolism.

Beta diversity analysis using Bray–Curtis dissimilarity revealed distinct differences in the GM composition across the study groups. At the baseline (day 0), the samples clustered tightly, indicating a relatively homogeneous microbial community structure among the participants prior to the intervention. In contrast, the samples collected after 30 days of either placebo or algae supplementation exhibited a broader distribution, reflecting increased interindividual variability in the microbial composition.

Interestingly, the group that received *H. elongata* presented a slight shift in the principal coordinates compared to their baseline values, indicating a potential change in the GM composition related to dietary intervention. However, because there was a partial overlap between the groups, this shift was not consistently observed across all the participants.

These results are consistent with the alpha diversity findings, which also revealed considerable variability at the individual level in response to algae. Taken together, these data suggest that while seaweed supplementation may influence the GM, its effects are more evident in some individuals than in others, highlighting the possibility of personalized responses to dietary interventions.

This type of person-to-person variability is increasingly recognized in the literature. For example, Johnson et al. [54] reported that microbial responses to daily dietary inputs varied widely among individuals, underscoring the need for personalized nutritional approaches. Similarly, Zeevi et al. [55] demonstrated that the glycemic responses to identical meals differed significantly across individuals, and these differences could be predicted based on the features of their gut microbiome.

#### 3.3.2. Evolution of Bacteria at the Phylum and Species Levels

**Figure 3:**
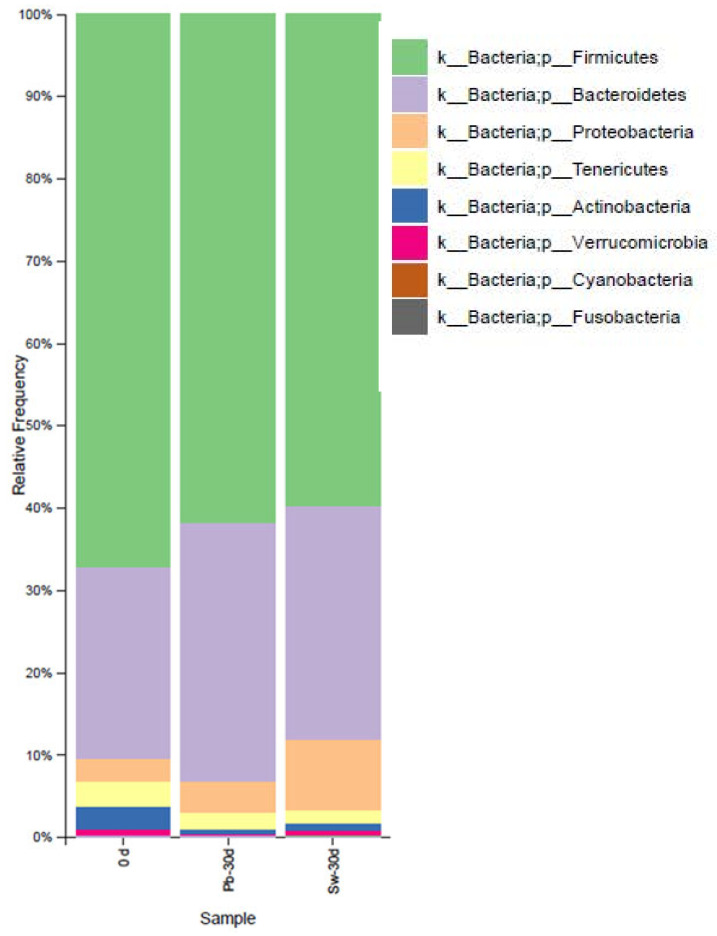
Average relative abundance of the gut microbiota at the phylum level in the two groups of volunteers before (0 d) and after treatment with placebo (Pb-30 d) or with seaweed (Sw-30 d).

Figure 3 shows the average relative abundance of the GM at the phylum level across all the participants at three different time points: before treatment (0 d), after 30 days of placebo (Pb-30 d), and after 30 days of seaweed supplementation (Sw-30 d).

The 16S rDNA sequencing data from the 10 volunteers revealed shifts in the composition of the dominant bacterial phyla. As expected, Firmicutes and Bacteroidetes remained the most abundant phyla in all the samples, which aligns with previous findings from human GM studies [56,57]. Interestingly, the relative abundance of Bacteroidetes slightly increased after seaweed intake (Sw-30 d) compared to that at the baseline and after placebo intake. There was also a small increase in the Proteobacteria levels in the seaweed group, which could reflect the selective effect of *H. elongata* intake on certain bacterial populations.

Other phyla, such as Tenericutes, Actinobacteria, Verrucomicrobia, Cyanobacteria, and Fusobacteria, were present in low proportions (<10%) and did not show marked variations between the groups. The microbial profile of the placebo group remained relatively similar to that of the baseline group, whereas the group that consumed seaweed showed a subtle trend toward increased relative diversity among the less abundant phyla. Although this is true, the data should be interpreted with caution, as interindividual variability in the GM composition is expected.

**Figure 4:**
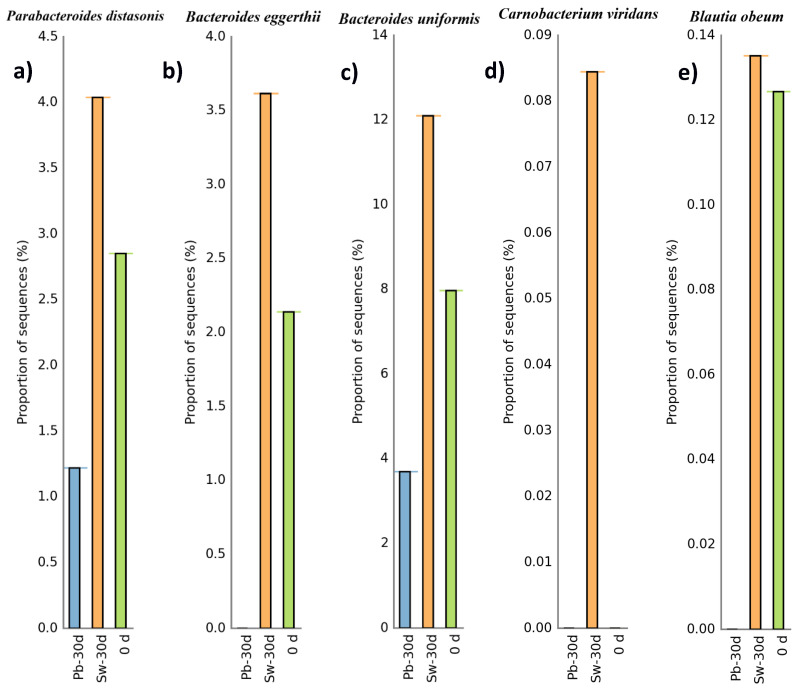
(**a**–**e**) Evolution of the relative abundance of different gut microbiota bacterial species involved in human health from day 0 to day 30 in the seaweed consumption group (Sw-30 d) and the placebo group (Pb-30 d).

Figure 4 shows the evolution of specific taxa from the baseline (0 d) to 30 d after seaweed (Sw-30 d) or placebo (Pb-30 d) supplementation. Figure 4a shows an increase in *Parabacteroides distasonis* abundance after 30 days of *H. elongata* intake, whereas its abundance decreased in the placebo group. The observed enrichment of *P. distasonis* aligns with the results of in vitro studies using the same seaweed and the findings on the prebiotic effects of inulin [58,59]. *P. distasonis* is known for its beneficial metabolic activities, including bile acid metabolism and SCFA production, and has been associated with improved metabolic health [60].

As shown in Figure 4b,c, there were increases in *Bacteroides eggerthii* and *Bacteroides uniformis*, respectively. Members of the Bacteroides genus are known for their important role in intestinal health, especially in the breakdown of complex carbohydrates and their contribution to the production of SCFAs and secondary bile acids, which help maintain the intestinal balance [61]. *B. uniformis*, in particular, has attracted attention for its ability to metabolize dietary polysaccharides and support the growth of other beneficial microbes, such as Lactobacillus, through crossfeeding [62]. These traits have been linked to anti-inflammatory effects and improved gut barrier function.

Interestingly, Figure 4d highlights the appearance of *Carnobacterium viridans* exclusively after 30 days of *H. elongata* supplementation, which was absent at the baseline and in the placebo group. While no studies to date have explored the health effects of *C. viridans* in humans, related species such as *C. divergens* and *C. maltaromaticum* are well documented for their use in food preservation. These strains have demonstrated antimicrobial activity, particularly against Listeria monocytogenes, and are known to increase the shelf-life and safety of meat and seafood products [63]. The postintervention presence of *C. viridans* could indicate that *H. elongata* creates a gut environment favorable to psychrotrophic lactic acid bacteria, although its specific role in the microbiome remains unclear and warrants further study.

Figure 4e reveals an increase in *Bacteroides obeum* as a consequence of *H. elongata* intake. Although this species has not been extensively studied in the context of inflammation, it belongs to a genus with known immunomodulatory effects. For example, *B. thetaiotaomicron* has shown anti-inflammatory benefits in animal models of Crohn’s disease [64]. Additionally, a meta-analysis by Zhou & Zhi [65] revealed that reduced levels of Bacteroides are common in individuals with inflammatory bowel disease, suggesting a protective role. Therefore, the increase in *B. obeum* could be relevant for modulating intestinal inflammation, although more research is needed to clarify its functional significance [66].

#### 3.3.3. Metabolic Pathways

**Figure 5:**
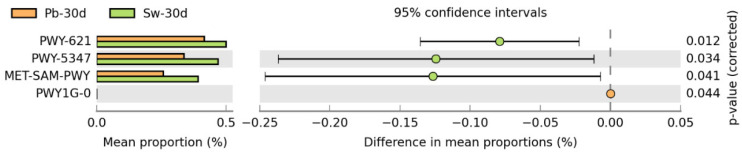
Differences in metabolic pathway proportions between the seaweed consumption group (Sw-30d) and the placebo group (Pb-30d). Only pathways showing statistically significant differences (*p* < 0.05) are displayed.

Metagenomic pathway prediction revealed significant changes in microbial functional potential after 30 days of *H. elongata* supplementation compared with the placebo (Figure 5). Three pathways showed a significantly greater abundance in the algae-supplemented group (*p* < 0.05): PWY-621, PWY-5347, and MET-SAM-PWY.

The PWY-621 pathway, which corresponds to sucrose degradation III (sucrose invertase) and is linked to the superpathway of L-methionine biosynthesis (transsulfuration), increased in abundance following seaweed consumption. This may indicate the enhanced microbial utilization of dietary sucrose-like substrates or seaweed–derived oligosaccharides, such as laminarin or mannitol, which resemble fermentable carbohydrates in structure [65]. Additionally, the association with methionine biosynthesis suggests the potential stimulation of amino acid metabolism, a process involved in microbial growth and host–microbe metabolic crosstalk [67].

The PWY-5347 pathway, which is involved in glycogen biosynthesis I from ADP-D-glucose, was also more active in the algae group. This pathway plays a role in intracellular energy storage, and its upregulation may reflect increased metabolic readiness or fermentative activity in response to the complex polysaccharides found in brown seaweed [68,69]. Enhanced glycogen production can be a marker of microbial resilience and adaptability to fiber-rich diets.

The MET-SAM-PWY pathway, which is responsible for S-adenosyl-L-methionine (SAM) biosynthesis, was also significantly upregulated. SAM acts as a universal methyl donor that is crucial for DNA methylation, gene regulation, and microbial communication [70]. The enrichment of this pathway could be indicative of increased biosynthetic and regulatory capacity in response to bioactive compounds present in *H. elongata*, including fucoidans and polyphenols, which have been shown to stimulate metabolic activity in gut symbionts [58].

### 3.4. Analysis of Short-Chain Fatty Acids

In this work, the concentrations of SCFAs in fecal samples collected before and after 30 days of supplementation with either *H. elongata* or a placebo were analyzed. The SCFAs measured included succinic, lactic, formic, acetic, propionic, isobutyric, butyric, isovaleric, and valeric acids. Overall, no statistically significant changes were observed in the concentrations of any of these metabolites in either group by the end of the intervention [71].

These results align with those of previous studies and suggest that the gut microbial response to specific dietary components, such as brown seaweed, can vary widely between individuals [72,73]. Although *H. elongata* contains potentially prebiotic compounds such as sulfated polysaccharides, the lack of detectable shifts in SCFA levels may reflect a few key factors. For example, the fermentability of these substrates may be limited among the dominant microbes present in our study population, or the dosage provided might have been too low to trigger a measurable response [74,75].

Importantly, SCFA concentrations in feces may not fully capture what is happening in the gut. Because most SCFAs are rapidly absorbed in the colon, especially in the proximal regions, fecal measurements may underestimate localized changes. Some studies have suggested that longer intervention periods or higher doses might be necessary to observe consistent shifts in SCFA profiles [76,77].

Finally, no adverse gastrointestinal effects were reported by any of the participants during the 30-day intervention period. Specifically, there were no complaints of abdominal discomfort, flatulence, bloating, diarrhea, or constipation in either the seaweed or placebo group. The treatment was well tolerated.

## 4. Strengths and Limitations of the Study

This study presents several strengths. To our knowledge, it is the first randomized, double-blind, placebo-controlled pilot trial in humans using whole encapsulated *H. elongata* to assess gut microbiota modulation. The use of high-throughput 16S rRNA sequencing, functional metagenomic prediction, and dietary assessment provides a multidimensional view of the intervention’s effects. Despite the small sample size, the double-blind design, strict inclusion criteria, and inclusion of a placebo control enhance the internal validity of the findings.

This study has several limitations that should be considered. First, the sample size was relatively small. Second, while a 30-day intervention can offer insights into short-term microbial responses, it may not be long enough to capture more sustained adaptations in the GM or significant changes in SCFA production [50,78]. When analyzed by the intervention group, the proportion of participants with obesity was slightly greater in the placebo group (60%) than in the *H. elongata* group (40%), whereas the proportion of normal-weight individuals was greater in the *H. elongata* group (40% vs. 20%). These baseline differences may have influenced the response of the GM to the intervention.

Another consideration is the use of self-reported dietary records. Despite our efforts to monitor intake carefully, these tools are subject to recall errors and underreporting, which could introduce bias and affect the interpretation of the results [79]. Finally, the general dietary patterns of the participants, characterized by a high intake of fat and protein and relatively low intake of carbohydrates and fiber, may have influenced the response of their microbiota to the algal supplements, which could attenuate the observable effects [80]. Future research should explore longer-term interventions and include diverse population groups to better understand the extent and sustainability of the effects of *H. elongata.* Studies integrating metagenomics and metabolomics could provide deeper insight into the functional changes in microbial communities and host responses. In addition, investigating the synergistic effects of *H. elongata* with other dietary fibers or prebiotic compounds may increase its potential as part of a comprehensive dietary strategy to improve gut health [81,82].

## 5. Conclusions

This randomized, double-blind, placebo-controlled trial is the first work evaluating the effects of whole encapsulated *H. elongata* on the GM of overweight individuals. Our findings suggest that a daily intake of 2 g of *H. elongata* for 30 days may benefit the GM composition by increasing the relative abundance of SCFA-producing bacteria, including *P. distasonis*, *B. eggerthii*, *B. uniformis*, and *B. obeum*. These changes occurred despite the participants’ overall suboptimal dietary patterns, characterized by high fat and low fiber intakes.

Although the intervention did not lead to statistically significant alterations in microbial diversity or SCFA concentrations, it did result in functional shifts at the metagenomic level, notably, the upregulation of the metabolic pathways related to carbohydrate metabolism and methylation. The heterogeneity in individual responses observed in both the taxonomic and functional analyses underscores the complexity of host–microbiota interactions and highlights the need for personalized nutrition strategies.

Given the limited number of participants, these results should be interpreted with caution and regarded as preliminary. The study was primarily designed to evaluate the feasibility and safety of *H. elongata* supplementation and to generate hypotheses for future research with larger samples. Taken together, our results support the potential of *H. elongata* as a prebiotic food ingredient that may contribute to GM modulation. Further studies with larger and more diverse populations, extended intervention periods, and integrated multiomics approaches are warranted to fully elucidate the health benefits and mechanisms of action of *H. elongata*.

## Figures and Tables

**Table 1:** Anthropometric data for each participant.

Participant	Group	BMI (kg/m^2^)	Waist Circumference (cm)
P1	Placebo	33.9	110.2
P2	Placebo	32.9	107.6
P3	Placebo	32.1	105.1
P4	Placebo	25.1	88.4
P5	Placebo	27.9	87.1
P6	*H. elongata*	26.3	86.5
P7	*H. elongata*	30.1	101.2
P8	*H. elongata*	27.2	95.4
P9	*H. elongata*	28.4	97.6
P10	*H. elongata*	30.5	102.5

**Table 2:** Energy and macronutrient intakes of the participants based on the 24 h dietary record.

	Mean	SD	Median	Q1	Q3	IQR
Water ^1^	2427.1	393.0	2361.7	2120.8	2699.8	2120.8–2699.8
Energy ^2^	1724.8	888.8	1463.4	1061.5	2401.1	1061.5–2401.1
Protein ^1^	83.8	35.2	69.0	64.4	95.6	64.4–95.6
Total fat ^1^	76.8	48.0	67.5	49.2	90.5	49.2–90.5
Carbohydrates ^1^	174.9	98.9	148.0	89.9	255.1	89.9–255.1
Sugars ^1^	70.6	41.5	56.4	46.7	66.5	46.7–66.5
Starch ^1^	104.3	77.4	89.2	43.6	127.3	43.6–127.3
Dietary fiber ^1^	17.2	9.9	18.0	9.6	19.2	9.6–19.2
Saturated fat ^1^	27.6	20.2	24.1	12.3	36.9	12.3–36.9
Monounsaturated fat ^1^	28.8	19.0	22.4	19.6	32.9	19.6–32.9
–	11.7	5.5	12.5	9.5	12.8	9.5–12.8
Cholesterol ^3^	257.7	170.4	249.0	130.9	414.6	130.9–414.6
Alcohol ^1^	0.0	0.0	0.0	0.0	0.0	-

^1^ grams (g); ^2^ kcalories (kcal); ^3^ miligrams (mg). Results are expressed as averages, standard deviations (SDs), medians, and interquartile ranges (IQRs).

**Table 3:** Energy and macronutrient intakes of the participants based on the 3-day dietary record.

	Mean	SD	Median	Q1	Q3	IQR
Water ^1^	2256.9	877.9	1988.1	1913.2	2971.1	1913.2–2971.1
Energy ^2^	1947.0	918.9	1734.4	1392.9	2143.1	1392.9–2143.1
Protein ^1^	100.0	39.6	93.1	73.2	107.7	73.2–107.7
Total fat ^1^	84.8	49.2	75.8	42.4	98.5	42.4–98.5
Carbohydrates ^1^	190.1	103.1	171.9	108.8	273.4	108.8–273.4
Sugars ^1^	67.6	55.0	44.4	32.9	65.2	32.9–65.2
Starch ^1^	122.6	69.2	119.7	63.4	158.5	63.4–158.5
Dietary fiber ^1^	14.7	5.6	14.9	10.7	18.7	10.7–18.7
Saturated fat ^1^	30.5	19.9	23.8	15.8	37.6	15.8–37.6
Monounsaturated fat ^1^	34.1	20.4	29.7	18.0	44.9	18.0–44.9
Polyunsaturated fat ^1^	11.8	7.5	9.5	5.0	16.0	5.0–16.0
Cholesterol ^3^	365.9	224.2	391.8	170.9	480.5	170.9–480.5
Alcohol ^1^	3.4	8.0	0.0	0.0	0.0	-

^1^ grams (g); ^2^ kcalories (kcal); ^3^ miligrams (mg). Results are expressed as averages, standard deviations (SDs), medians, and interquartile ranges (IQRs).

**Table 4:** Micronutrient (minerals and vitamins) and macronutrient intakes of the participants based on the 24 h dietary record.

	Mean	SD	Median	Q1	Q3	IQR
Calcium ^1^	1151.4	724.5	951.5	801.4	1058.1	801.4–1058.1
Iron ^1^	9.8	4.7	9.6	6.3	10.6	6.3–10.6
Iodine ^2^	342.0	210.7	350.5	185.0	499.7	185.0–499.7
Magnesium ^1^	295.8	93.4	253.2	247.5	330.7	247.5–330.7
Zinc ^1^	7.9	3.9	5.8	5.2	10.1	5.2–10.1
Natrium ^1^	2277.7	1043.3	1894.7	1495.6	2806.3	1495.6–2806.3
Potassium ^1^	2878.3	915.8	2456.6	2206.6	3656.8	2206.6–3656.8
Phosphorous ^1^	1581.8	540.2	1641.7	1308.8	1711.7	1308.8–1711.7
Selenium ^2^	83.5	34.1	98.7	68.2	106.4	68.2–106.4
Vitamin B1 ^1^	1.2	0.7	1.0	0.8	1.2	0.8–1.2
Vitamin B2 ^1^	1.7	0.8	1.5	1.3	1.5	1.3–1.5
Vitamin B3 ^1^	30.8	14.9	27.6	25.2	30.7	25.2–30.7
Vitamin B6 ^1^	1.6	0.3	1.5	1.4	1.9	1.4–1.9
Folic acid ^2^	147.6	65.0	129.4	93.6	187.1	93.6–187.1
Vitamin B12 ^2^	4.9	3.9	3.8	1.8	8.5	1.8–8.5
Vitamin C ^1^	73.7	30.4	68.7	57.0	91.3	57.0–91.3
Vitamin A ^2^	878.7	494.7	756.4	655.2	918.5	655.2–918.5
Retinol ^2^	485.0	338.6	533.4	257.5	738.2	257.5–738.2
Carotene ^2^	2442.1	2550.0	2188.4	182.3	4060.7	182.3–4060.7
Vitamin D ^2^	4.1	4.8	1.8	0.8	8.2	0.8–8.2
Vitamin E ^1^	5.5	3.6	4.3	2.8	7.4	2.8–7.4

^1^ miligrams (mg); ^2^ micrograms (µg). Results are expressed as averages, standard deviations (SDs), medians, and interquartile ranges (IQRs).

**Table 5:** Micronutrient (minerals and vitamins) and macronutrient intakes of the participants based on the 3-day dietary record.

	Mean	SD	Median	Q1	Q3	IQR
Calcium ^1^	944.5	505.3	831.9	775.9	831.9	775.9–831.9
Iron ^1^	11.4	5.2	10.1	8.8	10.1	8.8–10.1
Iodine ^2^	269.1	119.6	245.1	216.0	245.1	216.0–245.1
Magnesium ^1^	287.1	97.6	272.2	208.4	272.2	208.4–272.2
Zinc ^1^	12.1	5.9	12.7	8.9	12.7	8.9–12.7
Natrium ^1^	2973.2	1235.6	2629.2	2041.4	2629.2	2041.4–2629.2
Potassium ^1^	3134.0	1470.8	2943.8	2127.7	2943.8	2127.7–2943.8
Phosphorous ^1^	1518.5	566.5	1410.1	1090.8	1410.1	1090.8–1410.1
Selenium ^2^	98.2	59.8	87.5	58.5	87.5	58.5–87.5
Vitamin B1 ^1^	1.5	0.7	1.3	1.2	1.3	1.2–1.3
Vitamin B2 ^1^	1.6	0.8	1.5	1.1	1.5	1.1–1.5
Vitamin B3 ^1^	38.4	18.5	31.7	24.5	31.7	24.5–31.7
Vitamin B6 ^1^	2.0	1.0	1.6	1.4	1.6	1.4–1.6
Folic acid ^2^	179.5	75.2	167.3	129.3	167.3	129.3–167.3
Vitamin B12 ^2^	6.8	5.2	5.4	4.3	5.4	4.3–5.4
Vitamin C ^1^	86.4	56.9	79.9	38.3	79.9	38.3–79.9
Vitamin A ^2^	745.9	732.6	416.4	211.4	416.4	211.4–416.4
Retinol ^2^	245.1	198.6	226.1	129.7	226.1	129.7–226.1
Carotene ^2^	3239.8	4277.8	957.0	336.0	957.0	336.0–957.0
Vitamin D ^2^	3.6	4.5	1.6	0.5	1.6	0.5–1.6
Vitamin E ^1^	5.8	3.7	4.7	3.5	4.7	3.5–4.7

^1^ miligrams (mg); ^2^ micrograms (µg). Results are expressed as averages, standard deviations (SDs), medians, and interquartile ranges (IQRs).

## Data Availability

The original contributions presented in this study are included in the article.
